# Public–private collaboration in the provision of palliative care for children and adolescents with cancer: A Chilean experience

**DOI:** 10.1002/cnr2.1449

**Published:** 2021-05-29

**Authors:** Jazmine Fernández, Dunja Roje, Nuria Rossell, Marcela Zubieta

**Affiliations:** ^1^ Fundación Nuestros Hijos Santiago Chile

**Keywords:** Chile, palliative care, pediatric cancer, public–private

## Abstract

**Background:**

In Chile, children and adolescents with cancer in need of palliative care receive services through a collaborative scheme run in coordination between the hospitals of the public health system that attend children with cancer and the non‐profit civil society organization Fundación Nuestros Hijos (FNH).

**Aim:**

The main objective of this article is to offer a summary of the Chilean experience in the provision of palliative care services for children and adolescents with cancer, as an example of a public‐private partnership that improves the quality of life and the end‐of‐life experience for the children, adolescents, and their families.

**Methods and results:**

The palliative care program works with the children and their families as main members of the team, providing medical services for pain and symptom alleviation, psycho‐social support, rehabilitation for the improvement of quality of life, and aid to secure the best physical conditions for the child at home or in temporary housing for the whole family.

**Conclusion:**

The private‐public collaboration between the Chilean health system and the FNH is a successful model to help families suffering the devastating loss of a child.

## 1 BACKGROUND

According to the comprehensive definition of the World Health Organization (WHO), palliative care for children “is the active total care of the child's body, mind, and spirit, and also involves giving support to the family… Effective palliative care requires a broad multidisciplinary approach that includes the family and makes use of available community resources; it can be successfully implemented even if resources are limited”(p. 13).^1^ Pediatric palliative care (PPC) involves interventions aiming at the alleviation of symptom and psychosocial suffering and the improvement of the quality of life (QOL) of children and their families in every phase of a complex, chronic, and life‐threatening disease.^2^ This approach requires family and patient involvement through trusting communication with the health team and reliable support for homecare transitions if the child wishes so.^3^

In Chile, where about 500 children are diagnosed with cancer every year, 5‐year survival rates reach 78%. Still, 120 children will die either from advanced disease (80) or because of complications (40).^4^ Good outcomes in pediatric cancer treatment and good quality care have been possible, thanks to the decisive commitment of the Chilean Government in declaring cancer and palliative care as a public health priority concern. Thus, developed the corresponding health policies^5^ together with the public health insurance and strong alliances with NGOs to support all needs for children with cancer and their families. Currently, around 78% of children diagnosed with cancer are treated in public hospitals while the rest attends the private sector.^6^

Fundación Nuestros Hijos (FNH) is a nonprofit NGO supporting children and adolescents with cancer and their families through a comprehensive model with six programs: medical services, social services, hospital schools, rehabilitation, and palliative care. Mostly funded by private donors from civil society (individuals, business, and industry) and project‐specific national grants, FNH has its own rehabilitation center for children with cancer (CROFNH), operating as a clinic for outpatient services located in the vicinity of the main hospitals. FNH works in partnership with public (5) and private (2) hospitals that attend childhood cancer. Within this alliance, its programs reach 75% of the children and adolescent with cancer in Chile.

With the purpose of solving needs not covered by the public national services, FNH has developed a complementary program of palliative care, which includes the provision of medical supplies, services and equipment, psychosocial services, and transitional housing in a palliative care house for children or adolescents until 24 years old. The coordination with the pediatric oncology units in the hospitals is conducted by the specialized nurse who leads FNH's Medical Services Team.

## 2 SERVICES OF THE PPC PROGRAM

When a patient is in need of any FNH service (not only palliative care), the treating physician sends the request to the Medical Services Team. The PPC program offers medical and supportive care and psychosocial services provided as needed for each patient. The whole program is designed to operate fluently and timely, with the treating team and the family coordinating their needs directly without having to deal with bureaucratic procedures, paperwork, or institutional transfers or rules.

When a child presents disease progression with no possibilities for a cure, the treating team at the hospital coordinates the referral to PPC. The child continues a palliative care protocol at the hospital, and is also incorporated in the PPC of the FNH. The FNH's nurse coordinator participates directly in coordination meetings as well as clinical care. She is the initial contact with newly referred families and provides initial counselling as well as support in the follow‐up of the medical recommendations and prescriptions. Table 1 shows general data from FNH about the number of children who attended palliative care in 2019.

**TABLE 1:** Number of children attended in palliative care in 2019

	Number of children
Total children received	95
Male	57
Age 0–3 years	22
Age 4–7	24
Age 8–11	22
Age 12–16	22
>16 years	9
Length of PC	
<30 days	22
31–60 days	14
61–90 days	8
3 months–1 year	36
1–3 years	10
>3 years	4
Ambulatory and home care	91%
Home visits	37%
Palliative sedation	33%
Grieving counselling	60% (phone mainly)
Support with volunteers	87%

### 2.1 Medical supplies and services provided

Pain and respiratory distress are two of the most important symptoms that need to be under accurate care to provide comfort and QOL for the children in the PPC program. Opioids and all pain medication are secured by the health system and provided in the hospitals. However, public health services cannot provide medical equipment for pain relief and other symptoms at the patients' home. Thus, FNH complements such services. If needed, the families are provided with oxygen concentrators and/or aspiration pumps, and the parents are trained to use these confidently by the FNH's nurse coordinator, who also supports the training and supervision on analgesia. For children with deteriorating strength and mobility, who spend all or most of the time in bed, an antiulcers mattress is provided to prevent bedsores. For children with mobility limitations or impairments, wheelchairs suitable for their needs are provided to help their integration in family and social activities. Other services offered:

- *Loans of medical equipment*: Includes the loan of clinical cots, feeding pumps, portable oxygen, wheelchairs, among others. According to the needs, the equipment is given directly to the caregiver or delivered at their home, with monthly follow‐ups from a services assistant.
- *Medication*: A basic stock of medications (analgesics, antibiotics, antiemetics, gastric protectors, among others) that are not provided at the hospital are available to be given to the patients upon medical prescription.
- *Food supplements*: Including nutritional formulas that supplement with vitamins, minerals, protein, and fat, the nourishment of children, and adolescents under nutritional risk. Every food supplement must be prescribed by the treating physician.
- *Medical supplies*: A wide variety of items like disposable morphine pumps or catheters must be prescribed by the treating physician.
- *Integrative medicine*: available for patients and family members through FNH's volunteers who provide reflexology, Bach flowers, reiki, and biomagnetism in‐home visits.
- *Multidisciplinary home‐care visits*: The nurse coordinator together with other specialists according to the patients' needs to perform regular homevisits for the children attended in the main hospital of the Metropolitan Region.
- *Rehabilitation services*: All children in the PPC program are attended in the rehabilitation center (CROFNH) according to the proper examination and prescription of the physiatrist in charge. When necessary, these services are provided at home or in the hospital if the patient lives in the Metropolitan Region and is being treated in a partner hospital.

### 2.2 Psychosocial services

- *Transition home or palliative care home*: For children who live too far away from the capital city, and/or do not have the physical or economic condition for frequent traveling to the hospital, FNH provides a fully equipped house where the child and his/her family can live for the time they need.
- *House improvements*: A fund of up to USD$420.00 is available for families who need to perform reforms or modifications in their home to improve the care conditions for their child. These can be, for example, entrance modifications for better access and mobility in a wheelchair, bathroom conditions, other access, or hygiene‐related improvements.
- *Safe transfer*: The child and caregiver are provided with safe and comfortable transportation either by private FNH transportation or hired taxi services to all the medical appointments and treatments. In some cases, FNH supplies with a voucher for transportation.
- *Counselling*: By professionals and volunteers, the caregivers are provided with support and guidance in the process of empowerment that their role requires, to help them cope with decision‐making moments, while being self‐compassionate about their journey and their situation.
- *Wishes and recreation*: Managed mainly through volunteer teams, wishes are granted preferably at early stages when the child has the most of his/her capacities to fully enjoy the experience like visiting places, shows, meeting celebrities, etc.
- *Special situations*: Through volunteer teams, special needs are covered when necessary, like financial help for food requirements, clothing, and so on.
- *Mortuary fee*: A fund of up to USD $168.00 is available to help families with the expenses following the child's death.
- *Grieving parents group and workshops*: After 2 weeks of the child's death, the nurse coordinator makes contact with the family and invites them to participate in the workshop. This is an event scheduled four times a year in which the parents tell and listen to their experiences, share food, and are given a commemoration gift made by former participant parents.
- *Team's self‐care*: Building self‐care capacities in the team in order to prevent burnout is an important aspect of the work philosophy. Several activities are programmed yearly, from formal workshops to informal gatherings and recreational sharing of time outside of the workspace and schedule. It is the intention to implement a more structured self‐care program for the emotional and mental health of the FNH team.

### 2.3 Transition home

The palliative care house, or transition house, is a special service provided in the PPC program that has proven to be a pivotal component in the palliative care experience for many families. As the literature points out, most children in need of PC prefer to stay in their home instead of a hospital.^7^ One important barrier in many cases for the provision of domiciliary PC is the complexity of the specialized care that the child may need, requiring the permanent use of medical equipment. Other complex issues to overcome are the geographic distance, emotional distress, or socioeconomic problems of the family.

With the aim of providing family‐centered care, FNH equipped two houses where families of a child in PC can live as long as they need. In this house, the child and family have everything they need in order for them not to worry about equipment, transportation to the hospital, and so on. The house is built with the necessary space for special equipment and with the standards to keep biosecurity and all sanitary measures needed.

As long as the family lives in this house, the PC team is in contact with them. The nurse, psychologist, and social worker are in close contact to teach them essential care skills, provide emotional support, and help them build necessary support resources for the moment of their return to their own home when possible. The families can also decide to stay in this home until the child dies if that is their wish.

Volunteers from the PC team play an important role in daily life needs of the child and the family, offering company, play and crafts making, storytelling, trips and outdoor activities, integrative medicine, and spiritual support. If the child is also receiving care from the FNH Rehabilitation Centre, which is often the case, the specialists provide the therapy sessions in the house unless the child is in good physical condition to attend the center, in which case he/she and the caregiver are given transportation.

### 2.4 Transition home experience

One of the many examples that show how these services are integrated into the care of the children is the story of Esteban, a 13‐year‐old boy diagnosed with acute lymphoblastic leukemia. His parents and his two brothers (28 and 25 years old) were devastated with the diagnosis and determined to do whatever it was necessary for his treatment and health. They traveled from their 120 km distant home every time it was required for treatment. Unfortunately, Esteban relapsed twice and suffered an intracranial hemorrhage, which left him tracheostomized and with severe motor, visual, and speech impairments. He was referred to the PPC program. Although the family wanted to return home, they were extremely scared about all the procedures and care that they would have to do there, being so far away from the hospital. At this time, they were offered to stay in the transition house until they could feel safe enough to return home. During 4 weeks, the nurse and the rehabilitation team accompanied the parents in the procedures that they had to do until they felt safe. This meant that they would be sure that the procedures were correctly done but also that their role as loving and careful caregivers was empowering them, instead of weakening their confidence.

At the same time, FNH adapted the dining room of the family house in their hometown to become Esteban's room, where a clinical bed and everything he needed at home was prepared for him to be able to return. During his stay at his home, the PPC team from FNH and the hospital made weekly home visits until his death.

Two months after Esteban passed away, his parents started to attend the grieving parents' group of FNH, where they shared their story and talked about how important the time in the transitional house was for them, thanks to which they were able to give all the love and care that their son needed in their own home. Despite the tremendous family pain, they shared their experience with calm and peace, grateful that their son left in a serene way surrounded by all those who loved him so much.

## 3 FINAL COMMENTS

The private‐public collaboration between the Chilean health system and the FNH is a successful model to help families suffering a devastating loss from being overwhelmed with financial and bureaucratic problems that impede them from alleviating their child's suffering. It is important to remark that FNH has designed a program that complements clinical hospital care, adding benefits that allow families to meet their medical and financial needs. One of the most relevant aspects of this alliance is that it encourages the return of children and adolescents to their homes with their parents through strengthening the skills of caregivers and the delivery of medical and social services.

The transition house is the best example of the two main characteristics of the PPC program: on one side, it represents the effectiveness of the public–private collaboration between the health system and the FNH, where both institutions complement biomedical and psychosocial services. On the other side, the transition house enables the practice of the principles of family‐centered care, where all palliative care efforts take into consideration the emotional, financial, and social needs of the family.

Regardless of all the advances described, and although the model presented is efficient, it is necessary to make progress in expanding the availability of home care teams and the existence of transition houses in other parts of the country, with the aim of bringing services closer to people. This challenge will require the participation of the state, FNH, and other social organizations and all health establishments, particularly those primary care teams in each city.

## CONFLICT OF INTEREST

The authors have stated explicitly that there are no conflicts of interest in connection with this article.

## AUTHOR CONTRIBUTIONS

All authors had full access to the data in the study and take responsibility for the integrity of the data and the accuracy of the data analysis. *Conceptualization*, J.F., N.R., M.Z.; *Formal Analysis*, J.F., D.R., N.R.; *Writing—Original Draft*, J.F., D.R., N.R., M.Z.; *Writing—Review & Editing*, J.F., D.R., N.R., M.Z.; *Supervision*, D.R.; *Data Curation*, J.F.; *Investigation*, J.F.

## ETHICAL STATEMENT

Statistics in this manuscript are based on internal data of the FNH and do not involve patient's identification or other sensitive information. No ethical clearance was required.

## Data Availability

Data sharing is not applicable to this article as no new data were created or analyzed in this study.
